# Simultaneous Quantification of Multiple Urinary Naphthalene Metabolites by Liquid Chromatography Tandem Mass Spectrometry

**DOI:** 10.1371/journal.pone.0121937

**Published:** 2015-04-08

**Authors:** Daniel C. Ayala, Dexter Morin, Alan R. Buckpitt

**Affiliations:** 1 Department of Molecular Biosciences, School of Veterinary Medicine, University of California Davis, Davis, CA 95616, United States of America; 2 University of California Davis, Forensic Science, Graduate Program, 1909 Galileo Ct., Suite B, Davis, CA 95618, United States of America; Utah State University, UNITED STATES

## Abstract

Naphthalene is an environmental toxicant to which humans are exposed. Naphthalene causes dose-dependent cytotoxicity to murine airway epithelial cells but a link between exposure and human pulmonary disease has not been established. Naphthalene toxicity in rodents depends on P450 metabolism. Subsequent biotransformation results in urinary elimination of several conjugated metabolites. Glucuronide and sulfate conjugates of naphthols have been used as markers of naphthalene exposure but, as the current studies demonstrate, these assays provide a limited view of the range of metabolites generated from the parent hydrocarbon. Here, we present a liquid chromatography tandem mass spectrometry method for measurement of the glucuronide and sulfate conjugates of 1-naphthol as well as the mercapturic acids and N-acetyl glutathione conjugates from naphthalene epoxide. Standard curves were linear over 2 log orders. On column detection limits varied from 0.91 to 3.4 ng; limits of quantitation from 1.8 to 6.4 ng. The accuracy of measurement of spiked urine standards was -13.1 to + 5.2% of target and intra-day and inter-day variability averaged 7.2 (± 4.5) and 6.8 (± 5.0) %, respectively. Application of the method to urine collected from mice exposed to naphthalene at 15 ppm (4 hrs) showed that glutathione-derived metabolites accounted for 60-70% of the total measured metabolites and sulfate and glucuronide conjugates were eliminated in equal amounts. The method is robust and directly measures several major naphthalene metabolites including those derived from glutathione conjugation of naphthalene epoxide. The assays do not require enzymatic deconjugation, extraction or derivatization thus simplifying sample work up.

## Introduction

The incidence of lung disease including asthma and chronic obstructive pulmonary disease (COPD) has increased dramatically in the past 20 years and substantial evidence supports a role for vehicle emissions as a factor in the etiology of these diseases [1]. Vehicle emissions are a complex mixture containing a number of entities including polycyclic aromatic hydrocarbons, the most abundant of which are naphthalene and various methylnaphthalene derivatives [2].

Naphthalene is a volatile, lipid soluble polyaromatic hydrocarbon and an important component of various fossil fuels, cigarette smoke, and jet fuel (1–3% by weight) [3,4]. The finding that a number of low molecular weight polycyclic hydrocarbons such as naphthalene cause acute toxicity in lungs of mice raises the possibility that environmental exposures to these chemicals could pose a human health concern. Previous studies demonstrated that dose-dependent airway epithelial Clara cell (target cell) cytotoxicity due to naphthalene inhalation correlates strongly with high rates of metabolic activation by abundant, localized cytochrome P450 monooxygenases [5].

**Fig 1:**
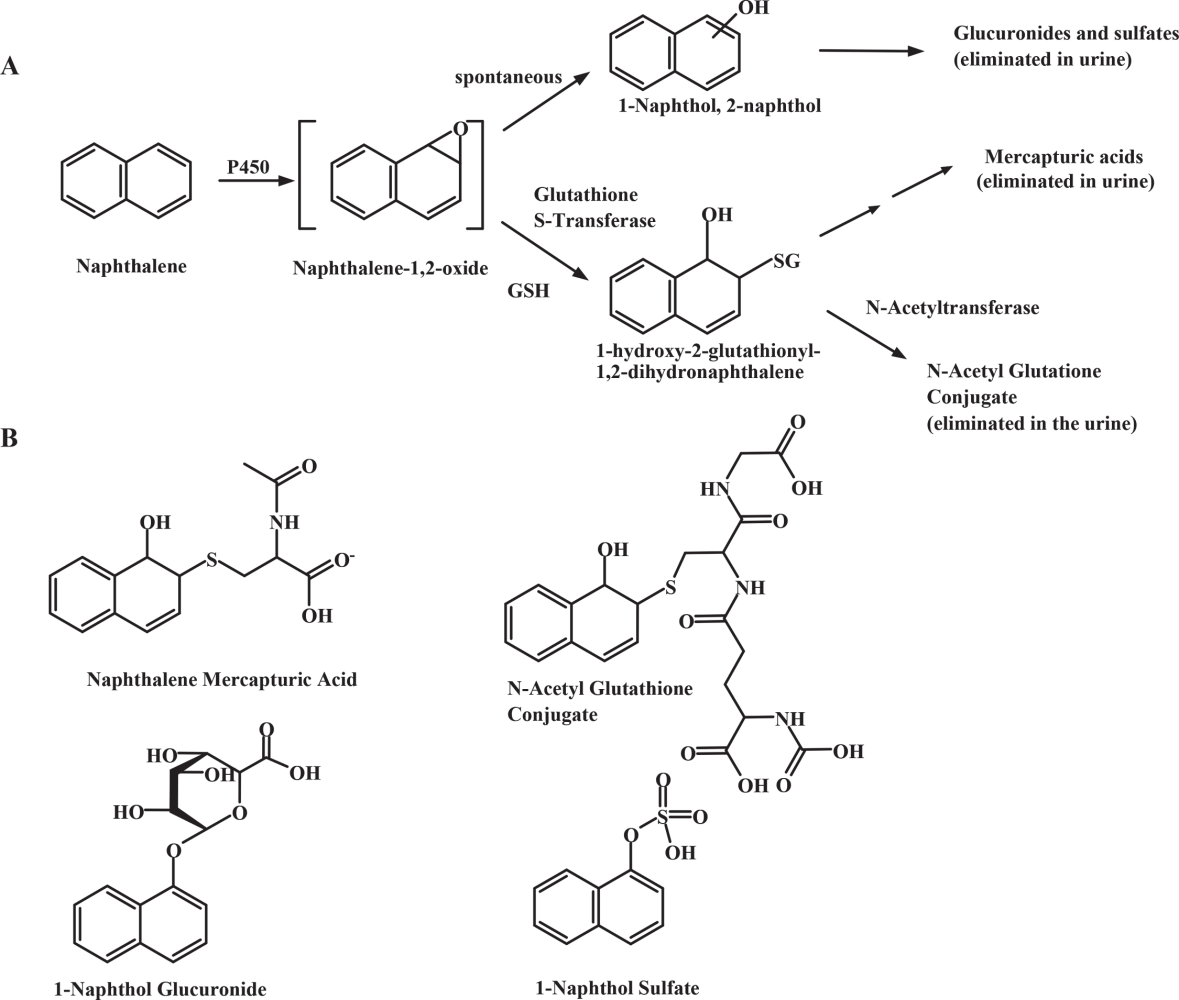
Overview of naphthalene metabolism and excretion. (A) Naphthalene metabolism showing the formation of both naphthol- and glutathione-derived urinary metabolites. (B) Chemical structures of targeted urinary naphthalene metabolites for LC/ESI-MS/MS analysis.

Several sensitive gas chromatography and gas chromatography-mass spectrometry methods have been developed as a means to effectively assess internal naphthalene exposures in industrial workers through detection of excreted naphthols [6,7]. Furthermore, in the NHANES (National Health and Nutrition Exposure Study) study Li and coworkers found detectable levels of urinary 1- and 2-naphthols in nearly the entire experimental cohort (general population, non-occupationally exposed) by GC-MS [8]. While excreted naphthols indicate naphthalene exposure and metabolism through the epoxide (Fig 1A), they underestimate the total amount of epoxide formed because they do not account for metabolites derived through the glutathione pathway. *In vitro* studies using human liver microsomes in the absence of glutathione and the GSH transferases demonstrated that the rates of dihydrodiol formation greatly exceed those of 1-naphthol [9]. In animal models, a significant proportion of urinary naphthalene metabolites are derived from the glutathione conjugates (Fig 1A) [10,11]. Yet, downstream metabolites of the glutathione adduct (mercapturic acids) have only been reported in humans using urine spot tests on paper chromatograms from individuals given large (500 mg p.o., approximately 1.4 mg/kg) doses of naphthalene [12]. Interestingly, other work in non-human primates failed to detect mercapturic acids of naphthalene but the reliability of this assay is uncertain because standards were not available [13]. In addition, an important consideration when using urinary naphthols to assess naphthalene exposure is that 1-naphthol excretion is also indicative of exposure to the insecticide, carbaryl [14]. Thus, there is a need to develop and validate quantitative methods for accurately measuring a range of naphthalene metabolites including the mercapturic acids, sulfate and glucuronide derivatives of naphthol, dihydrodiol, and any conjugates arising from naphthoquinones and the diol epoxide.

While many human exposure studies, including the NHANES assessments, have measured glucuronide and sulfate metabolites derived from naphthols in urine, very little attention has been given to other metabolites derived directly from the 1, 2-epoxide [15,16]. The work described here tested the hypothesis that the mercapturic acids and N-acetyl glutathione conjugates (N-acetyl GSH) formed from naphthalene oxide are quantitatively more important metabolites in mice than are the conjugates derived from naphthols. Since the glutathione-derived metabolites of naphthalene cannot arise from carbaryl, these metabolites may serve as better biomarkers of exposure to naphthalene than the naphthol-derived sulfate or glucuronide conjugates. Due to the highly polar nature of these conjugated metabolites as well as limited commercial availability of authentic standards, simultaneous quantification of multiple urinary metabolite targets presents a complex challenge. The availability of electrospray ionization (ESI) for mass spectrometry has allowed for development of both sensitive and specific LC-MS methods for a variety of compounds particularly for highly polar compounds that are not amenable to GC-MS analysis. Measurement of conjugates directly rather than after chemical or enzymatic hydrolysis decreases the time and complexity of analysis and allows separate measurements of sulfate, glucuronide and glutathione-derived naphthalene metabolites.

Accordingly, we developed a sensitive, robust LC-MS/MS method for the quantification of four major urinary naphthalene metabolites (Fig 1B). As proof of principle, we used the validated method to assess changes in the urinary metabolite profile in mice subjected to daily exposures to naphthalene at the OSHA short-term exposure limit (15ppm).

## Materials and Methods

### Animals

All animal work was conducted under protocols approved by the University of California—Davis Animal Use and Care Committee (PHS/NIH Assurance (A3433-01). Male Swiss-Webster mice (25-30g) were purchased from Harlan (San Diego, CA) and allowed free access to food and water. Animals were housed in HEPA-filtered isolators in an AAALAC accredited facility for at least one week before use.

### Chemicals and reagents

Naphthalene (scintillation grade) was purchased from Fisher Scientific (Waltham, MA). Deuterium-labeled naphthalene (d-8-99%) was purchased from Isotech through Sigma-Aldrich (Milwaukee, WI). N-Acetyl cysteine and glutathione were purchased from Sigma (St Louis. MO). All mobile phase solvents and solvents used to dilute samples were Optima grade purchased from Fisher Scientific (Waltham, MA). All other chemicals were reagent grade or better and purchased from Fisher Scientific (Waltham, MA).

### Naphthalene metabolite standards

Naphthol derivatives (1-naphthol-β-D-glucuronic acid and 1-naphthol sulfate) were obtained commercially (Carbosynth, San Bruno, CA and Research Organics, Cleveland, OH, respectively). 2-Naphthol glucuronide was prepared synthetically by oxidation of 2-naphthyl-β-D-glycopyranoside (Carbosynth) with 2,2’,6,6’-tetramethylpiperidine-1-oxyl (TEMPO, Sigma). The product was purified by semi preparative LC. 1,2-Naphthalene epoxides were synthesized as previously described [17]. Naphthalene mercapturic acid isomers were synthesized as follows: racemic naphthalene oxide (0.15–0.25 M) in ethanol was added drop wise with stirring to a 5 molar excess solution of N-acetyl cysteine dissolved in argon-sparged 0.1 M phosphate buffer at pH 8.5. The reaction mixture was stirred under argon for 4 h. The mixture was extracted with ether. The ether layer was discarded and the water layer partially evaporated to remove residual ether. The solution was acidified with acetic acid to pH 3, desalted using a SM-16 styrene-divinyl-benzene solid phase extraction cartridge (Biorad, Richmond CA), neutralized by the drop wise addition of pyridine, and lyophilized to dryness. The residue was reconstituted in 25% acetonitrile/water and purified by HPLC.

The N-acetyl glutathione conjugate of naphthalene was synthesized using the naphthalene glutathione conjugate. The glutathione conjugate (0.25 mmoles) was dissolved in 50 mL of argon sparged 0.1M Na_2_HPO4 buffer pH 8.5. The pH was carefully adjusted to pH 8.0 followed by the addition of 1 mmole Sulfo-NHS Acetate (Thermo Fisher/Pierce, Waltham MA), dissolved in nanopure water and added in one portion. The pH was immediately readjusted to pH 8.0. The reaction was allowed to proceed for 3 hours under argon. The reaction was then acidified to pH 3 with formic acid and applied to a SM-16 styrene-divinyl-benzene solid phase extraction cartridge (Biorad, Richmond CA) pre-equilibrated with 0.5% formic acid/water, and washed with 50 column volumes of 0.5% formic acid/water. The cartridge was then eluted with 25% acetonitrile and the eluent monitored at 254nm. The UV absorbing peak was collected and immediately neutralized to pH 6–7 with NH_4_OH. The solution was then lyophilized to dryness for 72 hrs under high vacuum to evaporate excess ammonium formate. The residue was dissolved in 25% acetonitrile/water and purified by preparative HPLC.

Purification of all synthetic metabolites was done by preparative HPLC using a 0.5% formic acid water/acetonitrile gradient system on a Phenomonex Sphericlone ODS2 column (5 μ, 22 x 250 mm) (Torrance CA). The HPLC purified peak was neutralized with pyridine and lyophilized. Samples were reconstituted as needed in 5% acetonitrile. Purity was assessed by LC-MS on a Thermo LCQ Advantage ion trap mass spectrometer (Thermo Fisher Scientific, San Jose CA). Metabolites were less stable in solution than as a powder and multiple freeze-thaw cycles of solubilized metabolites should be avoided.

### Internal standards

Deuterium-labeled naphthalene metabolites were prepared biosynthetically by treating mice with d-8 labeled naphthalene at relatively high doses and collecting the urine for use as a mixture of 99% d-8 labeled metabolites. Identical quantities of urine containing the d-8 metabolites were added directly to each of the unlabeled standards to generate standard curves and to urine from naphthalene-exposed animals to act as an internal standard for quantification of metabolites. Briefly, male Swiss-Webster mice (n = 4, body weight: 27.8–30.1 g) were placed (4 per cage) in glass metabolism cages (BioServe Biotechnologies, Laurel MD). The animals were dosed using a solution of 40 mg/ml 99% deuterium-labeled (d-8) naphthalene (dissolved in oleic oil) which was administered intraperitoneally at 0.05 ml/10 grams body weight (total dose of 200 mg/kg). The 24-hour urine was collected over dry ice. The urine was transferred to a clean Teflon tube and weighed to determine volume. An additional 2 volumes of acetonitrile were added to the urine and samples were stored at -20° C overnight. The next day, the urine was centrifuged at 9500*g* for 15min at 5°C. The supernatant was transferred to a Teflon bottle and lyophilized to dryness. The urine was reconstituted in 5% acetonitrile to the original urine volume, aliquoted and placed in storage at- 80º C. Metabolites in the urine samples dehydrate in acidic environments.

### Instrumentation

Composite urine samples (10 μl) were analyzed using a Waters 2795 Separations Module (Waters Corporation, Milford, MA). Naphthalene metabolite separation was carried out on a 2.1 x 150mm, 2.6μm Kinetex XB-C18 column (Phenomenex, Torrance CA) protected by a 2.1 x 2.0 mm C-18 SecurityGuard Ultra cartridge system (Phenomenex, Torrance, CA) maintained at a temperature of 35°C. The mobile phases were 0.1% acetic acid in H_2_O (A) and 0.1% acetic acid in 50% acetonitrile/water (v/v) (B). At a flow rate of 0.210 ml/min, initial conditions (90% A/ 10% B) were held isocratic for 14 min. Mobile phase B was increased by 5% (85% A/15% B) for 1 min followed by a linear gradient to 77% A/ 23% B at 65 min. Then mobile phase B was increased to 100% in 5 min. These conditions were held isocratic for 10 min (to 80 min). Finally, the mobile phase was returned to initial conditions (90% A/10% B) in 5 min. The column was allowed to re-equilibrate for 15 min. Total run time for each sample was 100 min.

MS/MS data were acquired using negative mode electrospray ionization (ESI-) on a Thermo LCQ Advantage ion trap mass spectrometer. Tandem MS parameters were optimized for all four metabolites (Table 1). While each metabolite had its own specific ion trap parameters and collision energies, the ESI source parameters were held constant throughout data acquisition. The optimized parameters for the ESI source were: source voltage set to 4.5 kV, capillary temperature at 250º C, nitrogen sheath and auxiliary gases at 40 and 10 (arbitrary units), respectively. Automatic gain control (AGC) was used to prevent accumulation of excess charge in the ion trap and maximum ion injection time was set to 200 ms during data acquisition. The trap was set to average three microscans during all targeted full-scan MS/MS acquisitions.

**Table 1:** Parameters for LC/ESI-MS/MS analysis of urinary naphthalene metabolites and internal standards (IS).

Metabolite	Retention time (min)	Molecular Ion (m/z)^a^	MS/MS fragments (m/z)^b^	CE^c^(%)
Mercapturic Acid	41.5	306	159, 288	45
N-Acetyl GSH	42.7	492	296, 314	42
Naphthol Glucuronide	68.4	319	113, 143, 175	32
Naphthol Sulfate	74.4	223	80, 143	32
d8-Mercapturic Acid (IS)	38.9	314	166, 295	45
d7-Naphthol Glucuronide	65.4	326	113,150,175	32
d7-Naphthol Sulfate	74.1	230	80, 150	32

^a^ Molecular ion, [M-H]^-^, acquired in negative ion ESI.

^b^To increase signal, product ion intensities were summed for quantification.

^c^Collision energy used to produce product ions.

Each metabolite had its own deuterium-labeled internal standard except for N-acetyl GSH, which was quantified using the internal standard for mercapturic acid. M/Z for the internal standard derived from naphthalene oxide (mercapturic acid conjugate) was +8 while those from naphthol (glucuronide and sulfate) were +7 AMU. Metabolite and internal standard peaks were identified by extracting targeted compound-specific fragment masses (m/z) from the total ion chromatogram (TIC) at the expected retention times. Thermo Xcalibur 2.0.7 software was used to control instrumentation and acquire raw MS data.

### Sample preparation

From each composite urine sample (pooled urine from three mice), 10 μl were transferred to a 0.5 mL microcentrifuge tube. Ten microliters of urine containing d-8 labeled metabolites (internal standard) were added in addition to 2 volumes (40μl) of Optima-grade acetonitrile. Samples were placed in the freezer at—80°C overnight. Samples were then thawed and diluted to 200 μl with 140 μl of 5% Optima grade acetonitrile/water. The samples were centrifuged at 16,000g for 30 min held at 4°C and the supernatant was transferred to an autosampler vial. A total of 10 μl of each sample was injected on column for LC-MS/MS analysis.

### Preparation of calibration and quality control (QC) standards

Stock solutions of all four metabolites were prepared by weighing and dissolving the compounds in 5% acetonitrile/water. These stock solutions were diluted further to prepare appropriate calibration and QC standards for analysis. Calibration standards were prepared by adding appropriate amounts of each metabolite solution into blank pooled urine taken from naïve mice. Final concentrations were 0.5, 1.0, 2.5, 5.0, 10.0, 25.0, and 50.0 μg/ml of urine for the mercapturic acid and 0.1, 0.25, 0.50, 1.0, 2.5, 5.0, and 10.0 μg/ml of urine injected for the N-acetyl GSH, naphthol glucuronide and sulfate.

For method validation, three QC standards (1.0, 4.0, 7.0 μg/ml for the mercapturic acid and 0.5, 2.0, 7.0 μg/ml for all other metabolites) were prepared by adding appropriate amounts of metabolite standard solutions into blank urine. Each calibration and QC standard contained differing amounts of each metabolite to closely mimic the natural variation found in urinary metabolite concentration during exposure experiments. All calibration and QC standards were prepared for LC-MS/MS analysis as described above.

### Specificity, sensitivity, and linearity

Specificity of the method was assessed by analyzing multiple blank pooled urine samples with the same LC-MS/MS parameters to ensure lack of mass signal in the same retention window as targeted metabolites or internal standards. The method limit of detection was estimated by multiplying the standard deviation of seven replicate measurements of the lowest QC standard by the Student’s t-value 3.143 (99% confidence level for seven replicates) for each metabolite. The method quantification limit was calculated as 10 times the standard deviation of the seven replicate measurements. Linearity of the method was assessed by plotting the peak area ratio of each metabolite/deuterated metabolite against the concentration spiked into control urine. The correlation between the peak area ratio and metabolite concentration was determined by least squares linear regression models. A full set of seven calibration standards were run in random order on four separate days to ensure that the method was robust and linear.

### Accuracy and Precision

Accuracy of the method was determined by calculating the percent bias observed in the replicate analysis of QC standards at three concentrations (n = 4 measurements) for each metabolite. Intra-day precision was determined by analyzing the three QC standards in triplicate (n = 3) during the same day and calculating the respective percent relative standard deviation (%RSD). Inter-day precision was expressed as the resulting %RSD from analyzing the three QC standards on four separate days. Due to the length of time between sample analyses, long-term autosampler stabilities of each naphthalene metabolite and respective deuterated internal standards were evaluated. This was done by analyzing calibration standard 3 (mercapturic acid, N-acetyl GSH, naphthol glucuronide, naphthol sulfate at 50.0, 10.0, 5.0, 1.0 μg/ml of urine injected, respectively) a total of five times over 63 hours and evaluating the percent bias and %RSD.

### Matrix effect evaluation and sample preparation recovery

To evaluate absolute matrix effect of the urine, duplicate sets of three QC standards (high, medium, low levels) were made up in solvent and in blank urine extract. The duplicate sets were analyzed in triplicate and area counts of each naphthalene metabolite were compared with the formula: Matrix Effect (%) = [Area counts (spiked urine))/Area counts (spiked solvent)]*100 [18]. Recovery of the entire sample preparation (precipitation and dilution) was characterized by analyzing duplicate sets of standard solutions (125 ng injected on column for the mercapturic acid, and 25 ng injected on column for all other metabolites) made up in both crude urine which was later extracted and post-extracted urine in triplicate. Ten microliters of internal standard urine was added to both sets of samples to test the suitability of the deuterium-labeled naphthalene metabolites for quantification. Calculated amounts per 10 μl injected were compared for each metabolite.

### Animal treatments and sample collection

Practical application of the method was determined by analyzing pooled urine samples from mice subjected to daily naphthalene exposures at levels relevant to human exposure limits. Animals were exposed to naphthalene vapors by inhalation in glass metabolism cages as described in detail previously [11,19]. Briefly, naphthalene vapors were generated by passing filtered, compressed air through a column containing crystalline naphthalene. This was mixed with compressed air in a mixing chamber; concentrations were monitored continuously by passing the samples through a UV flow cell monitored at a wavelength of 210 nm. Two groups of mice (3 per cage) were exposed to 15 ppm naphthalene for 4 hrs for 7 consecutive days. All exposures were concluded before 1 pm to avoid differences associated with the diurnal variations in glutathione levels. Following exposure, mice were transferred to a clean metabolism cage. All urine collection vessels were kept on dry ice so that urine was frozen immediately. Urine from the two groups of mice was collected at 24 hr intervals for a period of 7 days for a total of twelve composite samples (n = 2 samples for each day). As a control, two groups of three mice were exposed to filtered air for a total of seven days (n = 2 samples each day, 12 total samples). Urine was collected in the same way as from exposed animals. These samples were prepared for analysis as described in Section 2.5.

### Data analysis

To obtain the most meaningful urinary metabolite comparisons, calculated metabolite amounts per 10 μl injected on column were normalized against daily volumetric urine output and molecular mass resulting in nmoles of metabolite excreted per day. Daily urine output was determined gravimetrically using 1.00 as the estimated composite urine density. All statistical and data analyses were performed on Microsoft Excel 2010 and SigmaPlot ver. 11.

## Results

### Liquid chromatography mass spectrometry (LC-MS/MS)

**Fig 2:**
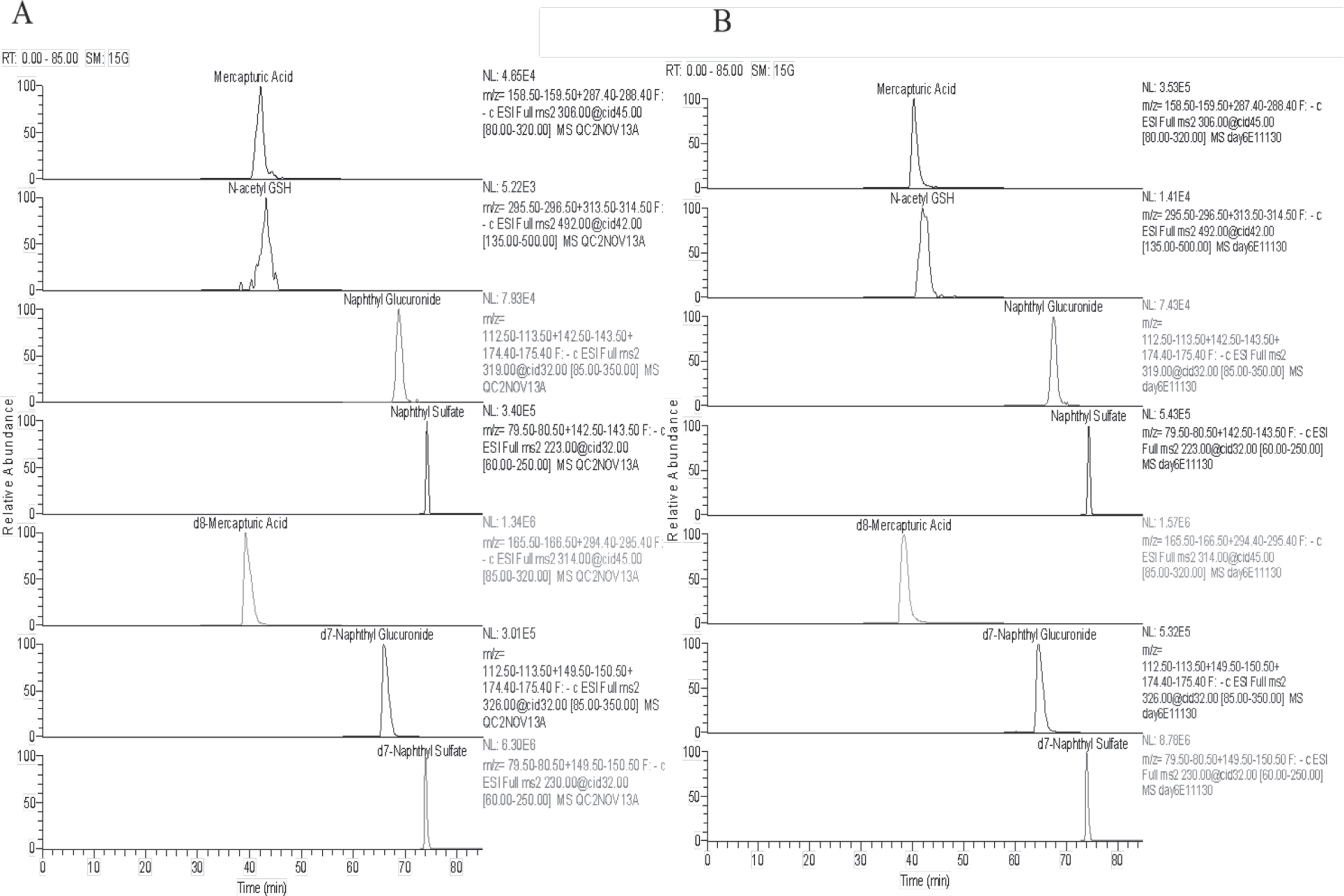
Extracted ion chromatograms of urinary naphthalene metabolites and deuterated internal standards. (A) QC Sample #2. Naphthalene mercapturate, N-acetyl glutathione conjugate, naphthol glucuronide, and naphthol sulfate at 40, 5, 70, 20 ng on column, respectively. (B) Pooled urine sample from day 6–7 of exposure treatments (n = 3 animals). Calculated amounts of each metabolite on column were: 124.8, 7.9, 45.3, 25.9 ng for mercapturic acid, N-acetyl GSH, naphthol glucuronide and naphthol sulfate, respectively.

We achieved optimal separation of all four major naphthalene metabolites and their corresponding deuterium-labeled internal standards with a 0.1% acetic acid water/acetonitrile gradient. All targeted metabolites eluted between 38.9 and 74.4 min (Table 1). Strong signals were observed for all metabolite-specific MS/MS fragments under all conditions tested. Fig 2 shows representative extracted ion chromatograms (EIC) of both a QC standard and typical composite urine sample from mice exposed to naphthalene. Two other metabolites were successfully separated, and were tentatively identified as the mercapturic acid conjugate derived from the dihydrodiol epoxide and dihydroxynaphthalene diglucuronide based on molecular ion (m/z) values 340 and 511, respectively (data not shown). Both were verified by high resolution mass spectrometry.

Authentic metabolite standards were not available so product ion spectra could not be compared, and accurate quantification could not be achieved.

### Specificity, sensitivity, and linearity

All analyzed matrix blanks (control urine from unexposed mice, n = 10) resulted in signals lower than the limit of detection for each metabolite and internal standard (S1A Fig). The method detection and quantification limits ranged from 0.9 to 3.4 and 2.9 to 10.8 ng on column, respectively (Table 2). We found that the estimated values were in general agreement with empirical values resulting from the lowest QC standards, except for the naphthol glucuronide. Our method reproducibly determined 5 ng injected on column (lowest QC) which is lower than the estimated method LOQ. S2A and S3A Figs. shows a representative chromatogram of each metabolite at the lowest QC level. Excellent, reproducible linearity (R^2^ range: 0.9930–0.9989) was achieved for the metabolite calibration ranges (Table 2). There were some unacceptable percent bias values from the lowest calibration standards (below the lowest QC levels) for each metabolite which was expected due to our estimated method LOD and LOQ values.

**Table 2:** Estimated limits of detection (LOD), quantification (LOQ), and linearity of LC-MS/MS method for urinary naphthalene metabolite analysis.

Metabolite	LOD (ng/10μl injected)	LOQ (ng/10μl injected)	Linearity	
			Range (ng/10μl injected)	Correlation Coefficient (R^2^)^a^
Mercapturic Acid	3.40	6.79	5–500	0.9989
N-Acetyl GSH	1.29	2.58	1–100	0.9930
Naphthol Glucuronide	2.30	4.60	1–100	0.9973
Naphthol Sulfate	0.91	1.82	1–100	0.9940

^a^ Average correlation coefficient (n = 5 calibration curves)

### Accuracy and precision

Accuracy and precision of the analyzed QC standards are shown in Table 3. Percent biases were all within ±15%, the intra-day and inter-day were within acceptable limits of 20% for all concentrations analyzed. The percent bias and percent RSD of the long-term autosampler stability study of calibration standard 3 ranged from 0.04–4.2% and 2.4–3.8% for all metabolites, respectively.

**Table 3:** Accuracy and precision results of quality control standards.

Metabolite	QC Standard	Nominal Amount (ng/10μl injected)	Determined Amount (ng/10μl injected)^a^	Accuracy (% bias)	Relative Standard Deviation (%RSD)	
					Intra-day (n = 3)	Interday (n = 4)
Mercapturic Acid	1	10.00	10.09	0.86	5.38	10.33
	2	40.00	41.25	3.13	5.20	16.15
	3	70.00	66.52	-4.97	16.87	6.08
N-Acetyl GSH	1	70.00	65.88	-5.88	8.58	1.32
	2	5.00	4.86	-2.84	10.14	4.58
	3	20.00	20.28	1.42	5.76	2.92
Naphthol Glucuronide	1	20.00	17.39	-13.07	2.59	7.48
	2	70.00	73.66	5.23	8.72	7.99
	3	5.00	4.65	-7.09	12.39	15.47
Naphthol Sulfate	1	70.00	70.84	1.20	3.54	0.77
	2	20.00	20.84	4.22	0.50	2.93
	3	5.00	4.54	-9.21	7.11	5.59

^a^ Average determined amount (n = 4)

### Matrix effect evaluation and sample preparation recovery

Calculated absolute matrix effects were between -16.8 to 11.8% for each metabolite at all concentrations analyzed (data not shown). The optimized urine sample preparation recoveries ranged from 86–101.6%.

### Method application

**Fig 3:**
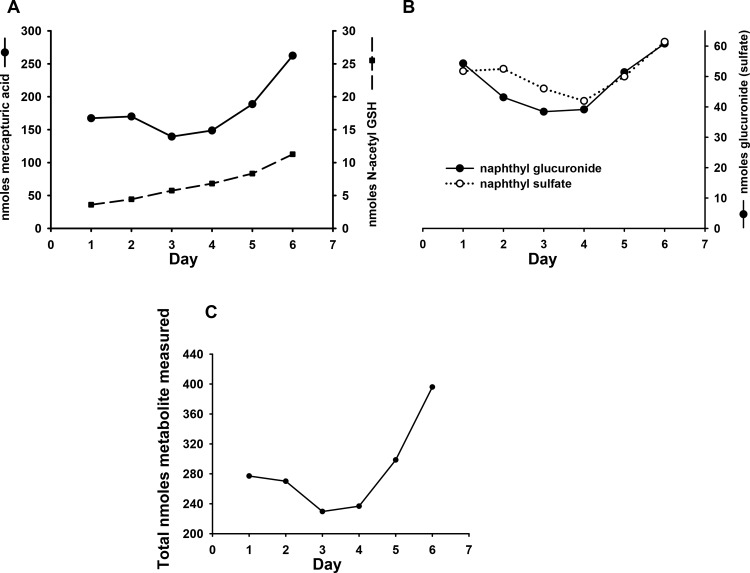
Urinary excretion of major naphthalene metabolites during repeated naphthalene exposure at the OSHA short-term exposure limit (15ppm x 4 hrs daily) via inhalation. (A) Average glutathione-derived metabolites excreted over entire exposure treatment (n = 2). (B) Average naphthol-derived metabolites excreted over entire exposure treatment (n = 2) (C) Total nmoles of quantified metabolite excreted over entire exposure treatment (n = 2). In all cases data represent the total nmoles of each metabolite excreted in a 24 hr period (ie concentration of metabolite (nmoles/ml) x mls urine collected for 24 hrs). Values are the mean of data obtained from 2 separate exposures (n = 2 cages) with 3 mice per cage.

**Fig 4:**
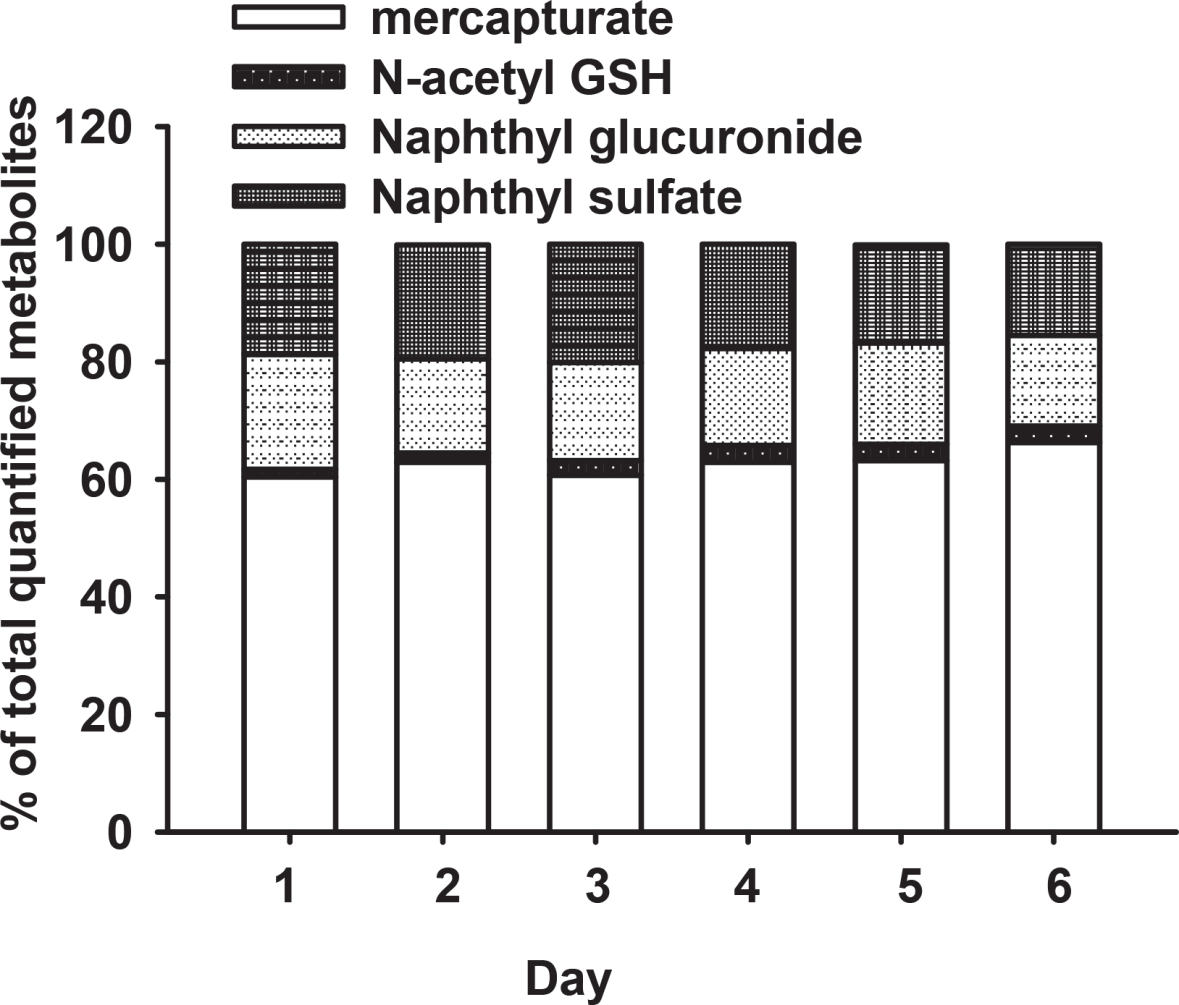
Naphthalene metabolite-specific percentages of total quantified metabolite during repeated naphthalene exposure treatments (15 ppm x 4hrs daily) via inhalation.

The entire experimental cohort (2 groups of 3 mice) produced daily urine samples which all contained detectable levels of all four targeted naphthalene metabolites. All control animal urine samples had levels below the method LOD. Fig 3A shows an overall increase in glutathione-derived metabolite (mercapturic acid, N-acetyl GSH) excretion over the entire exposure experiment. The mercapturic acid ranged from 139.5–263.5 nmoles excreted/24 hrs and represented, on average, greater than 60% of the total amount of quantified metabolites in each daily urine sample (range: 57.2–67.2%) (Fig 4). Over the 7 days, N-acetyl GSH steadily increased from 2.1 to 12.3 nmoles and represented an average 1–3% (range: 0.9–3.3%) contribution of total metabolite amount excreted (Fig 4).

The amounts of excreted naphthol glucuronide and naphthol sulfate ranged from 31.6–64.4 and 35.8–65.8 nmoles/24 hrs, respectively. Although the amounts of naphthol metabolites increased slightly over the 7 days of exposure (Fig 3B) with glucuronide and sulfate increasing by 12 and 19% on day 7 when compared to day 1, their percent contributions to total metabolite amounts fell slightly from 19.6 to 15.4% for naphthol glucuronide and 18.7 to 15.5% for naphthol sulfate (ranges: 21.5 to 14.6% and 20.8 to 15% for naphthol glucuronide and naphthol sulfate, respectively) (Fig 4). The amounts of glutathione- derived metabolites eliminated on day 7 were considerably greater than on day 1; the elimination of mercapturic acid and N-acetyl GSH increased by 57 and 213%, respectively (Fig 3A and 3B).

Fig 3C shows a decrease in total metabolite excretion during the second and third day with a subsequent sharp increase thereafter. This correlates well with the observed decrease in water and food intake and urinary output of both exposed groups observed during the second and third days of the exposure experiments (data not shown). Average total quantified naphthalene metabolites ranged from 178.5 nmoles during day 3 to up to 400 nmoles on the final day of exposure.

## Discussion

We present here a robust LC-ion trap tandem mass spectrometry method for the measurement of four major naphthalene metabolites in the urine of mice exposed to this PAH at levels relevant to human occupational exposure (15 ppm, OSHA STEL). The method shows good reproducibility and accuracy, and in comparison to the GC/MS methods for measuring 1- and 2-naphthol conjugates requires minimal sample cleanup [8]. The GC/MS methods for measuring naphthol conjugates entail hydrolysis, extraction, and derivative formation [20]. In addition, we were capable of differentiating metabolites which are rearrangement products of naphthalene oxide (glucuronide and sulfate derivatives of 1-naphthol) versus those generated by direct glutathione conjugation of the epoxide and excreted as mercapturic acids or as a novel N-acetyl glutathione derivative [21]. Conjugation with glutathione is one of the primary protective mechanisms against naphthalene cytotoxicity in animals [22] and, like other chemicals that undergo metabolic activation to generate soft electrophiles, naphthalene shows a glutathione threshold. Significant cytotoxicity and covalent protein adduct formation does not occur until glutathione is depleted substantially (20% of control GSH level) [22].

Although the 3D ion trap mass spectrometer has been used in the past as a quantitative detector [23,24], and under carefully controlled conditions can yield data that are comparable to a triple quadropole instrument [24], lack of sensitivity and relatively long chromatographic runs necessary to separate metabolites are distinct disadvantages of the use of this instrumentation. Nevertheless, using full-scan tandem MS and monitoring multiple fragment ions allowed us to verify the identity of the major peaks in our experimental samples to authentic standards by comparison of product ion spectra. In addition, the high concentrations of metabolites present in the urine of our dosed animals made the necessary method LODs and LOQs higher than the typical low ng/mL range found in environmental exposure studies. There was a significant increase in both signal and quality of the chromatography when using acetic acid versus other common LC-MS compatible mobile phase additives like formic acid and ammonium formate or acetate (data not shown). While small amounts of trifluoroacetic acid as a mobile phase additive resulted in the best chromatographic resolution, it strongly suppressed ionization in the negative mode. The targeted metabolites all have highly polar negatively-charged moieties (carboxylate, sulfate), thus were easily ionized using negative mode ionization.

Numerous studies measuring 1- and 2-naphthol conjugates in urine of exposed humans show slightly higher levels of 2-naphthol than 1-naphthol [15,16,25]. In contrast, we were unable to detect signals associated with 2-naphtholglucuronide in mouse urine using the 3D trap instrument. Further analysis of these urine samples using a Thermo Orbitrap XL mass spectrometer under identical chromatographic conditions revealed a signal for the 2-naphthol glucuronide. Here, the signal for the 2-naphthol derived conjugate was less than 10% of the signal associated with 1-naphthol glucuronide (data not shown). This is consistent with several of our earlier studies in mouse lung and liver preparations which show that 2-naphthol is generated at much slower rates than 1-naphthol (<10%). The underlying basis for these apparent differences is not clear but may be species-related. In other studies examining the metabolism of naphthalene in nasal tissues from the rhesus macaques, we have found that the ratio of 2- to 1-naphthol glucuronide is 1:2 (unpublished studies).

Our data (Tables 2 and 3) demonstrate that the method can reliably measure the four targeted urinary naphthalene metabolites with adequate sensitivity and reasonable accuracy and precision. With the exception of N-acetyl GSH, the metabolites yielded signal at the mid to high end of the standard curve. Thus, our method can assess metabolite levels of mice exposed to lower naphthalene levels by adjusting the dilution during sample preparation, though further validation studies are needed. The generation of deuterated internal standards biologically was straightforward and inexpensive and mitigated any excessive matrix effects from both the urine matrix (-16.8 to 11.8%), and sample preparation (protein precipitation and dilution (86–101%)).

Earlier work measuring 1- and 2-naphthol levels by GC/MS following enzymatic hydrolysis in a non-smoking control population reported levels (geometric mean) of these metabolites that are approximately 100 fold lower than the LOQ’s of the current method [26]. Thus, the current procedures would require modification to be useful in control human populations either through use of larger sample volume (1 ml) or through a more sensitive mass spectrometer. Naphthalene mercapturic acids are easily dehydrated in weak acid to generate the fully aromatic N-acetyl cysteine derivative of naphthalene so sample recovery from larger urine volumes on a solid phase extraction cartridge would have to properly buffer the solvent used to elute these metabolites.

The data in Fig 4 show that more than 60% of the metabolites measured in this work were derived from direct conjugation of naphthalene oxide with glutathione. A very small percentage of this total was from an N-acetyl glutathione derivative. This adduct has been reported previously in both mouse liver perfused with naphthalene and in mouse urine at 0.5 to 3% of a 50 mg/kg naphthalene dose administered intraperitoneally [21], percentages that are very similar to those reported here following inhalation exposures. The fact that we have not detected the N-acetyl glutathione derivatives in incubations of airway or nasal epithelial cells suggests that the excretion of this metabolite in the urine may be a function of metabolism in the liver followed by acetylation in the kidney. The N-acetyl glutathione metabolite is unusual and we are unaware of reports showing the elimination of this derivative from other chemicals that undergo metabolic activation.

Mercapturic acids are frequently reported metabolites in human urine derived from either exposure to exogenous electrophiles or through generation of electrophiles *in vivo*. These metabolites have proven to be very useful in establishing relationships between the incidence of lung cancer and exposure to volatile organic carcinogens such as 1,3-butadiene, acrolein and crotonaldehyde [27] as well determining the possible effectiveness of intervention strategies for aflatoxin-induced hepatocellular carcinoma [28]. With the exception of presumptive evidence showing the isolation of a mercapturic acid in the urine of human volunteers taking a relatively large dose of naphthalene (500 mg, p.o.), there have been no reports demonstrating the presence of this metabolite in urine of even relatively heavily exposed individuals such as cigarette smokers and fuel cell workers. In fact, studies in non-human primates given very large oral doses of naphthalene (up to 200 mg/kg) showed no increase in thioethers eliminated in the urine and no detectable depletion of hepatic glutathione levels. Both lung and liver of Rhesus macaques [29,30] and humans [31,32] metabolize naphthalene to the epoxide albeit at slower rates than rodents. Whether these rates are too slow to generate detectable amounts of mercapturic acid in urine or whether the amounts of epoxide formed are mainly metabolized to the diol and eliminated as a glucuronide or diglucuronide derivative of remains to be determined. Adaptation of the method described here provides a useful tool to address these issues.

During the course of the animal exposures, we noted an initial decrease in food and water intake in the first 2–3 days of exposure followed by increased levels of activity and parallel increases in water and food consumption. This corresponds with a greater than 15% decline in overall metabolite elimination in days 3 and 4 followed by a dramatic 25–30% increase. The proportion of the metabolites measured over this time period changed slightly with similar amounts of glucuronide and sulfate conjugates eliminated but with amounts of mercapturic acid and N-acetyl glutathione conjugate which increased by more than 40% from the initial levels in the urine. This is consistent with the dramatic increase in γ-glutamyl cysteine synthase noted in naphthalene tolerant animals [33].

## Conclusions

In summary, we present a method for quantitatively measuring 4 of the major naphthalene metabolites eliminated in the urine of mice exposed to naphthalene by inhalation and show that the most abundant metabolites measured are those arising from direct conjugation of naphthalene oxide with glutathione. In addition to the metabolites measured quantitatively, two other potentially important metabolites were observed, a diglucuronide of the dihydroxynaphthalene and N-acetyl cysteine derivatives of the diol epoxide. The unavailability of standards for these metabolites precluded their quantitative measurement and efforts are underway to prepare both metabolites synthetically. This method is currently being used to determine metabolite profiles in rats, where the nasal olfactory and respiratory epithelium plays a far more prominent role in the metabolism of naphthalene and in mEH and GST pi null animals. The method outlined here should be easily adapted to a triple quadrapole instrument in multiple reaction monitoring mode with a concomitant decrease in run times and increases in detection sensitivity.

## Supporting Information

S1 Fig Extracted ion chromatograms of targeted naphthalene metabolites in blank urine matrix. (PDF)

S2 Fig Extracted ion chromatograms of targeted naphthalene metabolites in low level QC standard. Calculated amounts of each metabolite on column were: 5.00 ng for naphthol glucuronide and naphthol sulfate.
(PDF)

S3 Fig Extracted ion chromatograms of targeted naphthalene metabolites in low level QC standard. Calculated amounts of each metabolite on column were: 10.0 ng for the mercapturic acid and 5.00 ng for the N-acetyl GSH conjugate.
(PDF)
