# Supplementary figures and images for: Simultaneous Quantification of Multiple Urinary Naphthalene Metabolites by Liquid Chromatography Tandem Mass Spectrometry
Source: PLoS One. 2015 Apr 8;10(4):e0121937. doi: 10.1371/journal.pone.0121937 (PMC4390350; doi:10.1371/journal.pone.0121937)

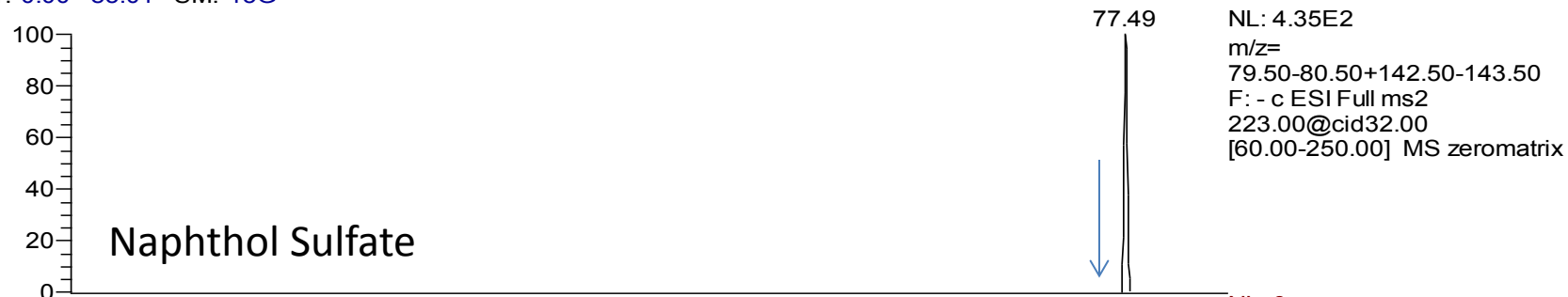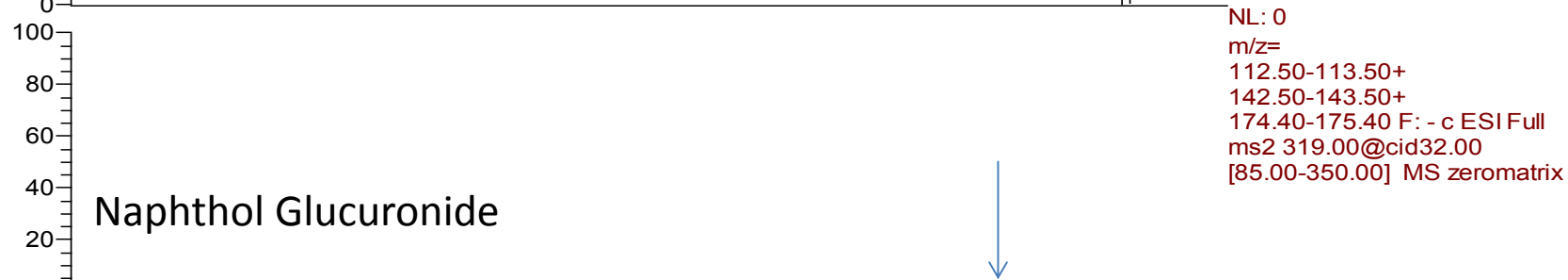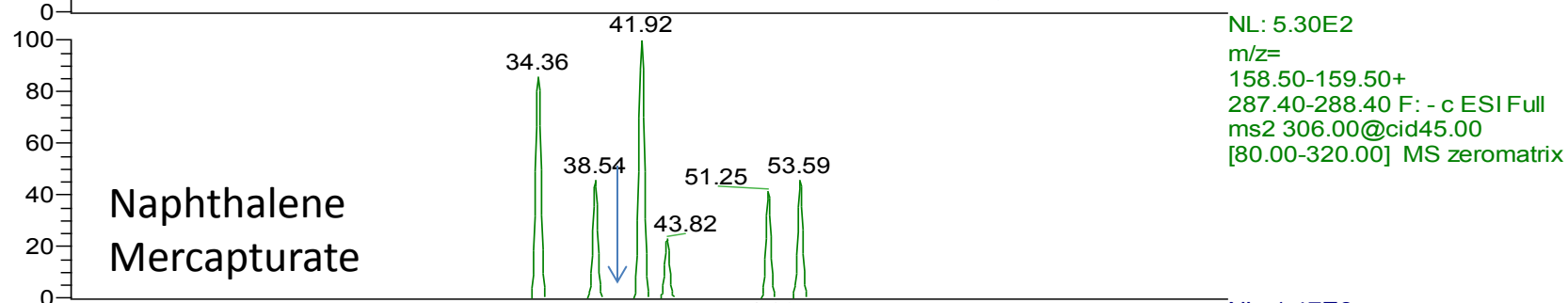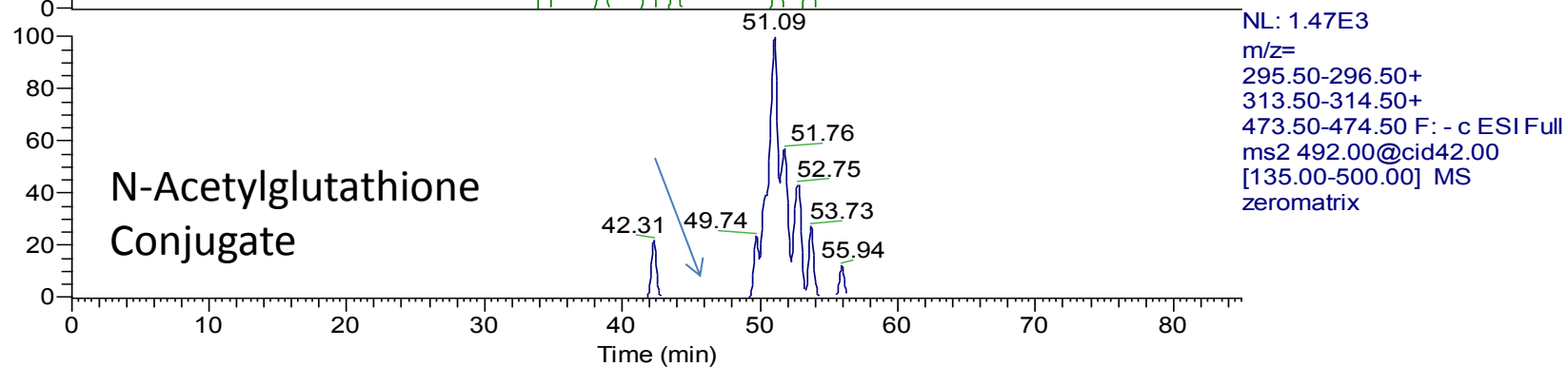

Supplement: S1 Fig — (PDF) [file pone.0121937.s001.pdf]
