# Supplementary material for: Simultaneous Quantification of Multiple Urinary Naphthalene Metabolites by Liquid Chromatography Tandem Mass Spectrometry
Source: PLoS One. 2015 Apr 8;10(4):e0121937. doi: 10.1371/journal.pone.0121937 (PMC4390350; doi:10.1371/journal.pone.0121937)
Supplement: S2 Fig — Calculated amounts of each metabolite on column were: 5.00 ng for naphthol glucuronide and naphthol sulfate. (PDF) [file pone.0121937.s002.pdf]

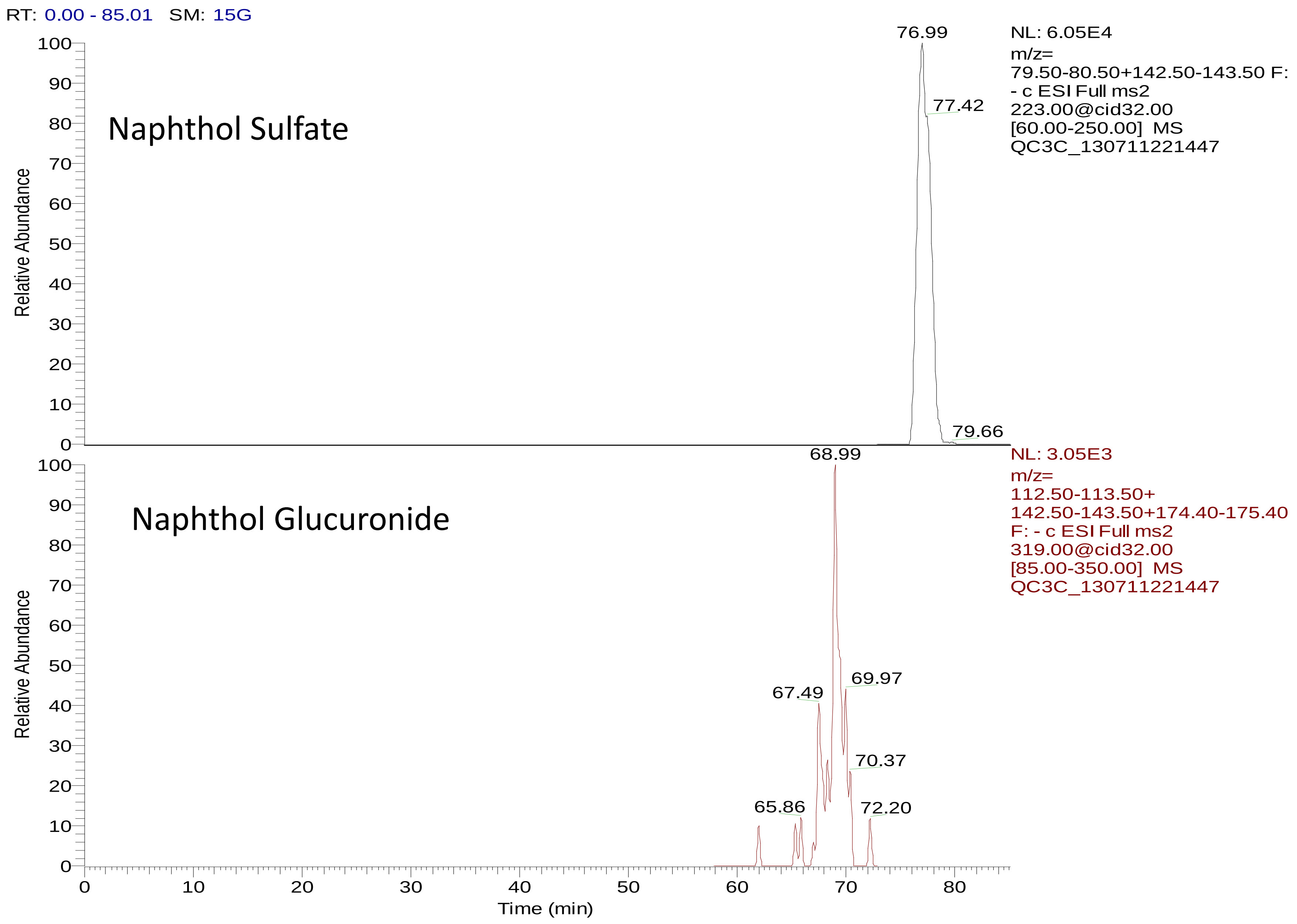
RT: 0.00 - 85.01 SM: 15G
Naphthol Sulfate
76.99
77.42
79.66
NL: 6.05E4
m/z=
79.50-80.50+142.50-143.50 F:
- c ESI Full ms2
223.00@cid32.00
[60.00-250.00] MS
QC3C_130711221447
Naphthol Glucuronide
68.99
67.49
69.97
70.37
65.86
72.20
NL: 3.05E3
m/z=
112.50-113.50+
142.50-143.50+174.40-175.40
F: - c ESI Full ms2
319.00@cid32.00
[85.00-350.00] MS
QC3C_130711221447
Relative Abundance
Time (min)
