# Supplementary material for: Simultaneous Quantification of Multiple Urinary Naphthalene Metabolites by Liquid Chromatography Tandem Mass Spectrometry
Source: PLoS One. 2015 Apr 8;10(4):e0121937. doi: 10.1371/journal.pone.0121937 (PMC4390350; doi:10.1371/journal.pone.0121937)
Supplement: S3 Fig — Calculated amounts of each metabolite on column were: 10.0 ng for the mercapturic acid and 5.00 ng for the N-acetyl GSH conjugate. (PDF) [file pone.0121937.s003.pdf]

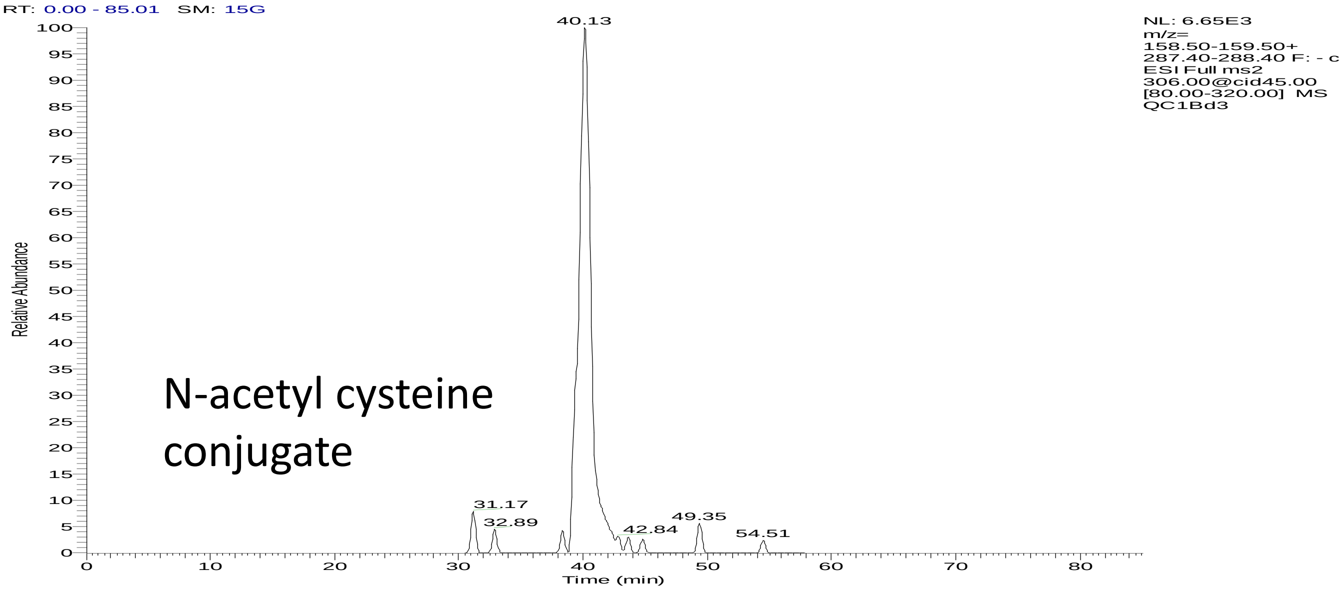

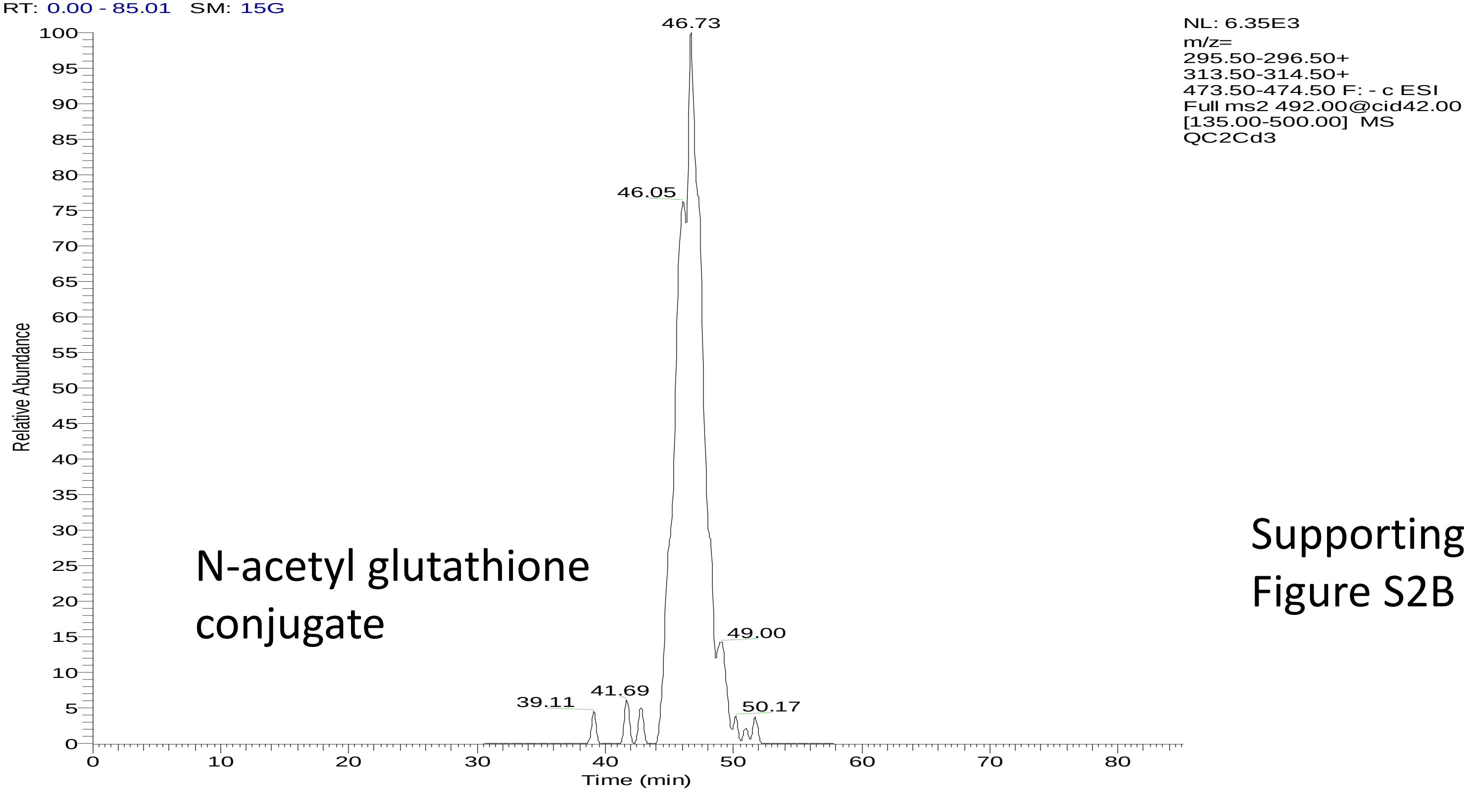

Supporting Information
Figure S2B
